# Supplementary material for: Cell Type-Specific Anti-Adhesion Properties of Peritoneal Cell Treatment with Plasma-Activated Media (PAM)
Source: Biomedicines. 2022 Apr 18;10(4):927. doi: 10.3390/biomedicines10040927 (PMC9032174; doi:10.3390/biomedicines10040927)
Supplement: Supplementary file 1 [file biomedicines-10-00927-s001.zip › biomedicines-1653462-supplementary.pdf]

Supplementary data

Cell type-specific anti-adhesion properties of peritoneal cell treatment with plasma-activated media (PAM)

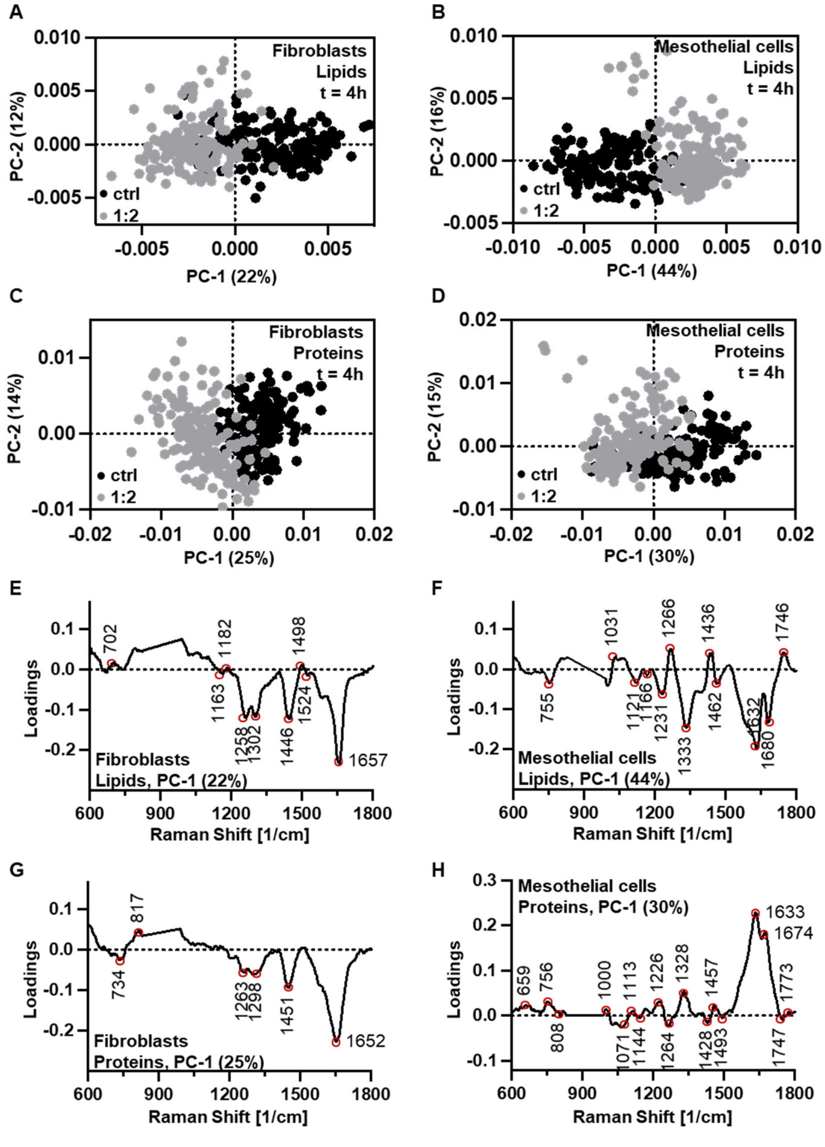

Figure S1. Characterization of cellular PAM effects by Raman imaging. (**A**–**D**) PCA based PC-1 vs. PC-2 score plot for the (**A,B**) lipid component and (**C,D**) the cytosolic protein component in fibroblasts and mesothelial cells. (**E**–**H**) Corresponding PC-1 loading plots for the (**E,F**) lipid component and (**G,H**) the cytosolic protein component in fibroblasts and mesothelial cells.

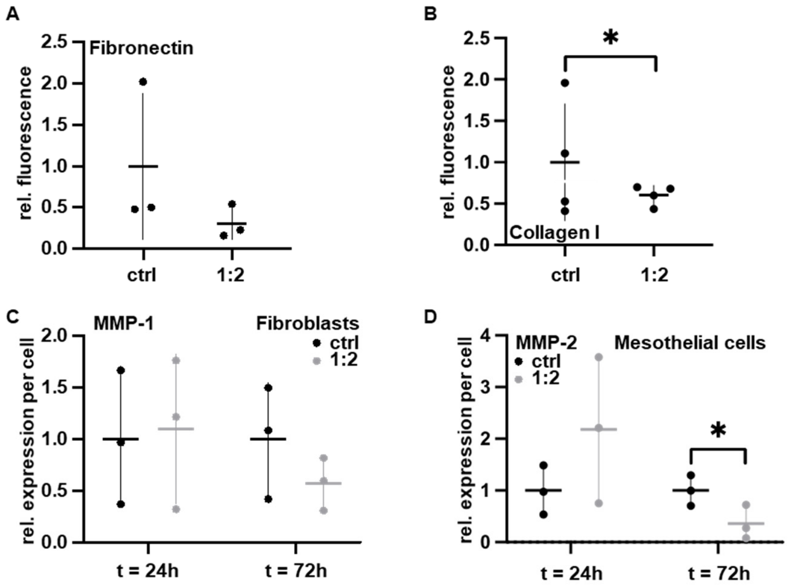

Figure S2. Fibronectin and MMP-1/2 expression analysis. Relative expression of fibronectin (**A**) and collagen I (**B**) per nucleus (mean intensity of (Fig. 4F,I) normalized to the control) of fibroblasts 120 h after 4 h of incubation with 1:2 diluted PAM (mean ± SD; *p < 0.05; paired t test). (**C,D**) Relative MMP-2 expression 24 and 72 h after 4 h of incubation with 1:2 diluted PAM relative to control (mean ± SD; *p < 0.05; paired t test).

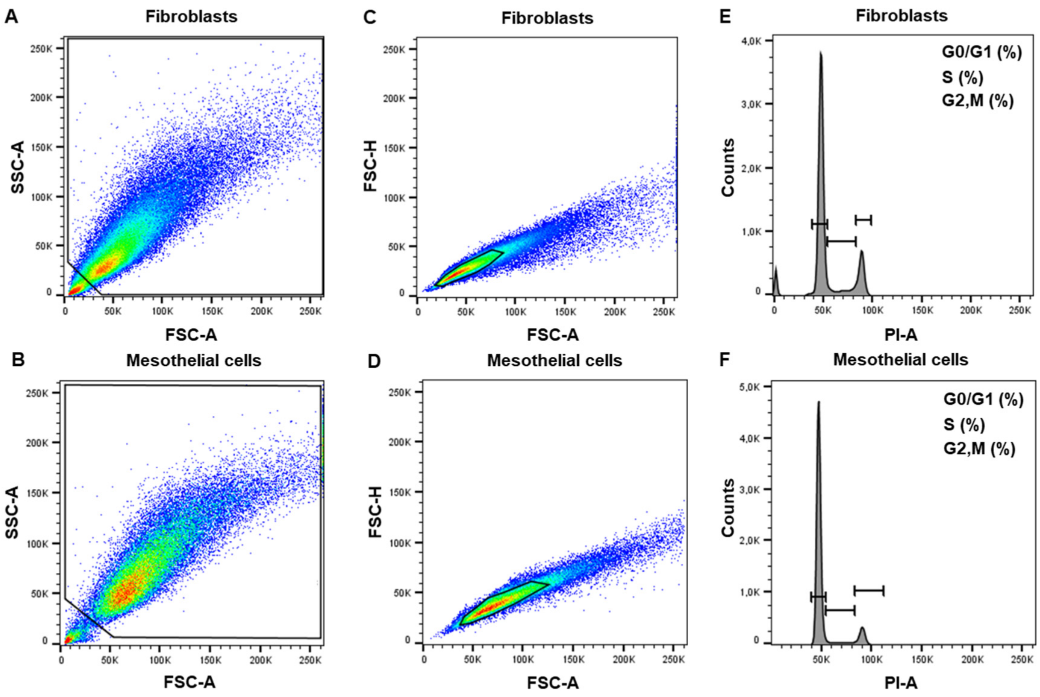

Figure S3. Gating strategy for flow cytometry. (**A**–**D**) Representative flow cytometry blots demonstrating the gating strategy for (**A,C**) fibroblasts and (**B,D**) mesothelial cells. Signals were measured and quantified using a FACSFortessa device (BD Bioscience) and FACS DIVA software v9 (BD Biosciences). Raw data were analyzed by FlowJo software v10 (FlowJo LLC). Forward- and side-scatter (FSC-H and SSC-H) characteristics were applied for dead cells and cell debris exclusion. Forward scatter area and height (FSC-A and FSC-H) characteristics were used to exclude cell doublets. (**E,F**) Representative DAPI histogram plot and setting of markers to quantitate the percentage of cells in each cell cycle phase.

Table S1: Identified Raman Peaks (cm^{-}) and their molecular assignment.

Peaks (cm^{-1})	Assignment	References
659	g-conformation methionine	[18]
690	Nucleotide conformation	[19]
702	Cholesterol, cholesterol-Ester	[20]
722	DNA	[21]
725	Adenine (DNA, RNA bases)	[19]
734	trans-conformation methionine	[18]
755	Tryptophane	[22,23,24]
785	Uracil, thymine, cytosine (DNA, RNA bases); O-P-O backbone	[19]
808	Nucleic acid backbone	[19]
915	Ribose	[19]
996	C-O ribose, C-C bond	[25]
1000	Phenylalanine; NADH	[26]
1063	C-C stretching mode	[27]
1068	PO_2^- stretching DNA/RNA	[28]
1071	Glucose	[20]
1113	Amide III and different groups of proteins	[29]
1121	C-C lipids, fatty acids	[18]
1163	Tyrosine (collagen type I)	[19]
1182	Cytosine, guanine, adenine	[22]
1226	Amide III	[28]
1231	Amide III	[25]
1249	Amide III	[25]
1258	Amide III	[22]
1263	Amide III, -CH in proteins	[19,26,28]
1266	Proteins	[19,24]
1300	-CH	[30]
1302	CH_2 twisting or wagging mode in phospholipids, $-CH_3$ und $-CH_2$ wagging or bending in lipids	[19,24]
1328	-CH	[30]
1333	-CH	[30]
1428	C=OO in aspartate and glutamine, $-CH_2$ in proteins	[18,31]
1436	$-CH_2$ scissoring in lipids; -CH in lipids and proteins	[27,28]
1443	Guanine, adenine (DNA, RNA bases); -CH deformation (DNA, RNA)	[28]
1446	$-CH_2$ bond and deformation in lipids	[22,23,32]
1451	$-CH_2$ deformation	[33]
1457	Deoxyribose	[34]
1462	CH_2 in lipids	[18]
1493	Amid II (C-N bond; N-H bond)	[25]
1498	Amide II	[25]
1520	Amide II (C-N)	[25]
1555	Amide	[35]
1632	Amide I band (C=O)	[36]
1633	Amide I band proteins (C=O)	[19,25,36]

1652	Amide I (proteins)	[37]
1657	Fatty acids, triacyl glyceride	[19,25,38]
1659	Amide I	[39]
1674	Amide I band (proteins)	[37]
1746	C=O lipids	[22]
1747	-OH amino acids (aspartate, glutamine)	[18]
1749	Amide I (C=O)	[40]
